# GnRH agonist trigger in focus: does protocol choice between PPOS and antagonist strategies affect outcomes?

**DOI:** 10.1007/s00404-025-08229-7

**Published:** 2025-10-29

**Authors:** Yusuf Aytaç Tohma, Fazilet Kübra Boynukalın, Meral Gültomruk, Mustafa Bahçeci, Gürkan Bozdağ

**Affiliations:** 1 Department of Infertility, Bahceci Ankara IVF Center, Ankara, Turkey; 2 Department of Infertility, Bahceci Fulya IVF Center, Istanbul, Turkey; 3 Department of Research & Development, Bahceci Fulya IVF Center, Istanbul, Turkey

**Keywords:** Progesterone primed ovarian stimulation, GnRH antagonist, GnRH agonist trigger, Oocyte yield

## Abstract

**Purpose:**

The aim of this study was to assess the effectiveness of the GnRHa trigger in the PPOS and GnRH antagonist protocols when using a GnRH agonist (GnRHa) trigger.

**Methods:**

This retrospective cohort study conducted at Bahçeci Fulya IVF Center (January 2022–January 2024) included 802 patients undergoing ovarian stimulation with a starting dose of 300 IU gonadotropins using either a GnRH antagonist (*n* = 372) or PPOS protocol (*n* = 430), followed by a GnRHa trigger. The primary outcome was oocyte yield; secondary outcomes included pregnancy rates following the initial frozen embryo transfer (FET).

**Results:**

Baseline characteristics, including female age, BMI, and infertility duration, were comparable between groups. Although PPOS was associated with a shorter stimulation duration [10 (9–11) vs. 10 (10–11) days, *p* = 0.002], lower progesterone levels on trigger day [1.3 (0.74–1.48) vs. 1.5 (0.83–1.63) ng/ml, *p* = 0.002], and higher LH levels [4 (1.89–5.2) vs. 3.3 (1.4–4.1) IU, *p* < 0.001], oocyte yield and embryological outcomes were similar (*p* > 0.05 for all). Clinical pregnancy rates [63.6% vs. 63.8%, *p* = 0.95] and live birth rates [51.7% vs. 52.2%, *p* = 0.87] were also comparable. Regression analysis identified embryo quality (*p* = 0.003), but not stimulation protocol (*p* = 0.766), as a significant predictor of live birth.

**Conclusion:**

PPOS and GnRH antagonist protocols provide similar oocyte yield and live birth rates following GnRHa trigger. These findings indicate that progestin use in PPOS is not associated with inferior clinical outcomes in the setting of GnRHa trigger; however, the underlying mechanisms and long-term efficacy require further investigations.

## What does this study adds to the clinical work?

**Table:** 

This study demonstrates that GnRHa trigger yields similar oocyte numbers and live birth rates in both PPOS and GnRH antagonist protocols. These findings support the use of PPOS as a clinically effective alternative, offering scheduling flexibility without compromising reproductive outcomes—despite the unclear mechanism of pituitary suppression by progestins.	

## Introduction

Gonadotropin-releasing hormone (GnRH) antagonists are widely used during ovarian stimulation (OS) to prevent a premature luteinizing hormone (LH) surge and subsequent luteinization [1]. This is achieved through competitive inhibition at the GnRH receptors in the pituitary gland [2]. However, it is associated with disadvantages that includes high cost, being a cold chain drug and the necessity of daily administration [3]. Therefore, in recent years, there has been a shift to protocols that are easier to use and more cost-effective such as Progestin-Primed Ovarian Stimulation (PPOS).

PPOS, a method that suppresses the pituitary using oral progestins administered concurrently with gonadotropins and continued until the ovulation trigger, may serve as an option for pituitary suppression during ovarian stimulation (OS) in segmented IVF cycles [4]. While oral progestins are known to suppress the LH surge [5], the mechanisms by which progesterone interacts with estradiol to regulate the LH surge remain unclear. The proposed mechanism of action for progesterone involves a reduction in the frequency of GnRH pulses [6]. Nevertheless, PPOS has quickly become IVF practices given its practical advantages, such as being easier to use and more cost-effective compared to alternative methods, especially in the context of segmented cycles.

In segmented cycles, triggering with gonadotropin-releasing hormone agonists (GnRHa) reduces the incidence and severity of ovarian hyperstimulation syndrome (OHSS), particularly for high responders [7, 8]. Although the GnRH a trigger protocol is preferred in segmented IVF cycles due to reporting low pregnancy rates and high early pregnancy loss rates due to an insufficient luteal phase when a standard luteal phase support was used in fresh embryo transfer [9, 10], some clinicians might also have concerns about the risk of a suboptimal response to GnRHa trigger, resulting in a suboptimal oocyte yield. Although heterogeneity exists and the suboptimal response might be defined by one of the following criteria: (i) a post-trigger luteinizing hormone (LH) level of less than 15 mIU/ml, (ii) a reduced total number of oocytes retrieved, or (iii) a decreased oocyte yield [11], calculated as the ratio of the total number of oocytes retrieved to the total number of follicles larger than 10 mm on the day of triggering, there is no universal consensus on this subject.

Accordingly, this study aimed to assess the effectiveness of the GnRHa trigger in the PPOS and GnRH antagonist protocols, which utilize distinct mechanisms for preventing premature LH surge. The comparison was based on oocyte yield and pregnancy outcome parameters.

## Materials and methods

This retrospective cohort study was conducted at Bahceci Fulya IVF Center between January 2022 and January 2024. It was approved by the Institutional Ethics Board (application number: 158). Its inclusion criteria were i) female age of 19–45 years, ii) utilization of GnRH antagonist or PPOS protocol iii) preference of GnRHa for triggering oocyte maturation and iv) cases with starting daily gonadotrophin dosage of 300 IU. The exclusion criteria were i) severe male factor (sperm concentration lower than 2 × 10^6^/ml), ii) utilization of GnRHa for LH suppression and iii) application of dual trigger for oocyte maturation.

A total of 802 patients were included who underwent OS with either the GnRH antagonist protocol (*n* = 372) or the PPOS protocol (*n* = 430), using GnRHa trigger. The analysis focused on the first FET cycles (*n* = 689) resulting from these OS cycles, comprising 329 patients in the GnRH antagonist group and 360 patients in the PPOS group.

### OS regimen

#### GnRH antagonist protocol

The recombinant follicle-stimulating hormone (rFSH; Gonal-F; Merck Serono, Germany) and/or purified human menopausal gonadotrophin (hMG; Merional; IBSA, Italy) was initiated on the second or third day of menstruation with 300 IU. The GnRH antagonist (0.25 mg cetrorelix; Cetrotide; Merck Serono, Germany) was started when the follicle diameter reached 13 mm or estradiol concentration was > 300 pg/mL and was continued until the day of final oocyte maturation. Whenever the diameter of at least two follicles had reached ≥ 18 mm, final oocyte maturation was triggered by administering 0.2 mg of triptorelin.

#### PPOS protocol

Ten mg of medroxyprogesterone acetate (MPA; Tarlusal; Deva, Turkey) was commenced daily on the second or third day of menstruation and continued until the day of final oocyte maturation. Initial fixed daily gonadotropin dose of 300 rFSH (Gonal-F; Merck Serono, Germany) or hMG (hMG; Merional; IBSA, Italy; Menopur, Ferring Pharmaceuticals, Saint-Prex, Switzerland) was administered. Whenever the diameter of at least two follicles had reached ≥ 18 mm, as measured on TV-USG, final oocyte maturation was triggered by administering 0.2 mg of triptorelin.

### Endometrial preparation

For estrogen priming, either an incremental oral estrogen regimen (Estrofem, Novo Nordisk, Turkey) or a continuous regimen was utilized. The incremental protocol consisted of 4 mg/day on days 1–4, 6 mg/day on days 5–8, and 8 mg/day on days 9–12. Transvaginal ultrasonography (TV-USG) was performed between days 10 and 13 of the cycle to assess endometrial thickness.

When the endometrial thickness exceeded 7 mm and serum progesterone (P4) levels were below 1.5 ng/mL, daily intramuscular (IM) progesterone (Progestan, Koçak Farma, Turkey) at a dose of 50–100 mg or daily 50 mg subcutaneous (sc) progesterone (Prolutex, IBSA, Lugano, Switzerland) was initiated. Embryo transfer was scheduled on day six of progesterone administration**,** blastocyst-stage embryo transfer was planned.

Oral estrogen supplementation and luteal phase support with daily IM or sc progesterone were maintained until the ninth week of pregnancy.

Oocyte retrieval, denudation, intracytoplasmic sperm injection (ICSI), embryo culture, vitrification, and warming procedures were performed as previously described by us [12].

### Outcome measures

The primary outcome was the number of oocytes collected as might be defined as an oocyte yield. The secondary outcome parameters were numbers of usable embryos, and clinical and live birth pregnancy rates after initial frozen embryo transfer (FET). Usable blastocysts were those with sufficient morphological quality for freezing and assessed by established grading systems, whereas clinical pregnancy rate (CPR) was defined as the detection of an intrauterine gestational sac via TV-USG per ET, live birth rate. (LBR) was defined as the number of deliveries beyond 24 weeks of pregnancy per ET. Miscarriage was defined as the loss of clinical pregnancy before the gestational week of 12.

## Results

There were no significant differences between the GnRH antagonist (*n* = 372) and PPOS (*n* = 430) groups in terms of female age, body mass index (BMI), and infertility duration (Table 1). The distribution of infertility diagnoses was also comparable between the groups. The duration of ovarian stimulation was significantly shorter in the PPOS group [10(9–11) vs 10(10–11) days, *p* = 0.002], while serum progesterone (P4) levels on the trigger day were significantly lower in PPOS group [1.3(0.74–1.48) vs [1.5(0.83–1.63) ng/ml, *p* = 0.002]. Additionally, the LH levels on the trigger day were significantly higher in the PPOS group [4(1.89–5.2) vs 3.3(1.4–4.1) IU, *p* < 0.001]. No statistically significant differences were observed between the two groups regarding cumulus–oocyte complex count (COC), mature (MII) oocytes, two pronuclei (2PN) embryos, maturation rate, fertilization rate, blastocyst development rate, or the number of frozen blastocysts (*p* > 0.05 for all comparisons) (Table 1).

**Table 1:** Comparison of patient, endocrinologic, and embryologic characteristics

	GnRH antagonist (372)	PPOS (430)	*p*
Female age	29(26–32)	30(27–33)	0.636
BMI	25.9(22.7–28.9)	25.9(22.3–28.7)	0.971
Duration of infertility	4(2–5)	4(2–5)	0.058
Infertility diagnosis (*n*/%)			0.812
PCOS	137(36.8)	151(35.1)	
Male infertility	108(29)	132(30.7)	
Endometriosis	14(3.8)	17(4)	
Tubal factor	5(1.3)	6(1.4)	
Unexplained	108(29.1)	124(28.8)	
Duration of stimulation	10(10–11)	10(9–11)	0.002
Trigger day E2	4662(2749–5556)	4640(2682–5715)	0.42
Trigger day P4	1.5(0.83–1.63)	1.3(0.74–1.48)	0.002
Trigger day LH	3.3(1.4–4.1)	4(1.89–5.2)	< 0.001
COC	19(14–24)	19(14–24)	0.371
MII	16(11–19)	16(11–19)	0.799
2PN	12(8–16)	12(8–16)	0.923
Maturation rate	80.3(72.3–91.3)	82.1(75–92.3)	0.158
Fertilization rate	78.1(70–90)	79.2(72.7–90.9)	0.146
Blastulation rate	41(25–57)	43(29–57)	0.142
No of frozen blastocyst	5(2–7)	5(3–8)	0.213
Cancelation rate	39(10.5)	46(10.7)	0.92

Values are presented as median (interquartile range) or number (percentage), as appropriate. *p* values represent comparisons between GnRH antagonist and PPOS groups. Statistical significance was set at *p* < 0.05

The outcomes of the first FET cycles of the included patients were analyzed. There were no significant differences between the GnRH antagonist (*n* = 329) and PPOS (*n* = 360) groups in terms of female age, BMI, number of embryos transferred (single or double), or the presence of PGT-A (Table 2). Similarly, embryo quality distribution (good, moderate, or poor) was comparable between the two groups (*p* = 0.185). CPR was similar between the GnRH antagonist (210/329, 63.8%) and PPOS (229/360, 63.6%) groups (*p* = 0.95). Likewise, LBR showed no significant difference (*p* = 0.87) between the GnRH antagonist (172/329, 52.2%) and PPOS (186/360, 51.7%) groups. The miscarriage rate was 18.1% (38/210) in the GnRH antagonist and 18.7% (43/229) in the PPOS group** (***p* = 0.67**).** Similarly, the multiple pregnancy rate was 6.1% (13/172) and 11.3% (21/186) in the GnRH antagonist and PPOS groups, respectively (*p* = 0.31) (Table 2).

**Table 2:** Comparison of the outcomes of the first FET cycles

	GnRH Antagonist (*n* = 329)	PPOS (*n* = 360)	*p*
Female age	29(27–32)	30(27–32)	0.665
BMI	25.9(22.4–29)	25.8(22.4–28.3)	0.629
No of embryos transferred			0.99
Single	286/329(83.9%)	303/360(84.2%)	
Double	53/329(16.1%)	57/360(15.8)	
PGT-A			
Presence	35/329(10.6%)	46/360(12.7%)	0.383
Absence	294/329(89.4%)	314/360(87.3%)	
Embryo quality			0.185
Good	287/364(78.8%)	304/417(73%)	
Moderate	69/364(19%)	102/417(24.4)	
Poor	8/364(2.2%)	11/417(2.6%)	
Clinical pregnancy	210/329(63.8%)	229/360(63.6%)	0.95
Live birth	172/329(52.2%)	186/360(51.7%)	0.87
Miscarriage rate	38/210(18.1%)	43/229(18.7%)	0.67
Multiple pregnancy	13/172(6.1%)	21/186(11.3%)	0.31

Values are presented as median (interquartile range) or number (percentage), as appropriate. *p* values represent comparisons between GnRH antagonist and PPOS groups. Statistical significance was set at *p* < 0.05

The linear regression analysis evaluating the factors influencing the number of usable frozen blastocysts indicated that female age (*B* = −0.062, *p* = 0.025) and presence of male factor infertility (*B* = −0.709, *p* = 0.005) are significant negative predictors of the number of frozen embryos. In contrast, the number of COC (*B* = 0.204, *p* < 0.001) is a strong positive predictor. Other factors, including BMI (*p* = 0.906), stimulation protocol (PPOS vs GnRH antagonist) (*p* = 0.095), and total gonadotropin dose (*p* = 0.537), did not show a significant impact (Table 3).

**Table 3:** Linear regression analysis evaluating the factors influencing the number of usable frozen blastocysts

	Beta	*P*
Female age	−0.073	0.025
BMI	0.003	0.930
Male factor (presence/absence)	−0.091	0.005
Protocol GnRH antagonist vs PPOS	0.053	0.095
Total gonadotrophin dosage	−0.020	0.537
COC	0.438	0.000

Binary logistic regression analysis was conducted to evaluate the predictors of live birth. As might be expected, embryo quality (good, moderate, or poor) was significantly associated with live birth (*p* = 0.003). Compared to good-quality embryos, poor-quality embryos had a lower likelihood of live birth (OR = 0.344, 95% CI 0.113–1.044, *p* = 0.060), and moderate-quality embryos also had a reduced likelihood (OR = 0.545, 95% CI 0.372–0.797, *p* = 0.002). The stimulation protocol (PPOS vs. GnRH antagonist, OR = 0.955, 95% CI 0.705–1.293, *p* = 0.766) also showed no significant effect (Table 4).

**Table 4:** Binary logistic regression analysis of live birth

Variables	OR (95%CI)	*p*
Female age	1.01 (0.97–1.05)	0.815
BMI	0.98 (0.95–1.01)	0.242
Male factor	1.03 (0.74–1.43)	0.848
Protocol^1^	0.96 (0.71–1.29)	0.366
COC	0.99 (0.98–1.02)	0.873
No of embryos transferred	1.47 (0.94–2.3)	0.92
ET day^2^	0.94 (0.37–2.38)	0.138
Embryo quality^3^	0.34 (0.11–11.04)	0.003
PGT-A^4^	0.75 (0.45–1.26)	0.276

^1^refers to comparison of GnRh antagonist vs PPOS

^2^refers to comparison of day 5 vs day 6

^3^refers to comparison of good-quality embryos vs others

^4^refers to comparison of presence and absence of PGT-A

## Discussion

In this study, we compared PPOS protocol and the GnRH antagonist protocol, both followed by a GnRHa trigger, with respect to oocyte yield and pregnancy outcomes. Since the suppression mechanism in the PPOS protocol is not yet clearly defined, data from our study are valuable as they address two key questions on this topic. The first question is whether the GnRH agonist trigger is as effective in the PPOS protocol compared to the GnRH antagonist protocol with regard to oocyte yield**.** The second question is whether the pregnancy outcomes of embryos resulting from GnRHa trigger stimulation is comparable when generated after an ovarian stimulation with PPOS or GnRH antagonist protocol. The results obtained from this study’s data indicate that oocyte yield is not adversely affected in the PPOS protocol following GnRH agonist trigger, and the pregnancy outcomes derived from the resulting embryos are not inferior to those of the GnRH antagonist protocol.

When comparing PPOS and GnRH antagonist protocols, it is evident that they involve different hormonal profiles during ovarian stimulation. Specifically, on the trigger day, the LH level in the PPOS group was significantly higher than the GnRH antagonist group, indicating a statistically significant difference between the two protocols. Although endogenous LH levels were significantly higher on the trigger day in PPOS cycles against antagonist cycle, their effectiveness was similar between the two protocols. The number of oocytes retrieved, the maturation rate, and the number of blastocysts obtained were similar between the two protocols and those findings are consistent with the findings by Kalafat et al. that had been reported previously [13]. The precise mechanism by which progestins suppress pituitary function remains incompletely understood. Dozortsev et al. proposed that sustained exposure to progestins may lead to depletion of LH reserves and thereby inhibiting a spontaneous LH surge [14]. The depleted LH reserves may lead to incomplete LH surge which may lead to insufficient oocyte yield. The effectiveness of GnRHa trigger in flexible PPOS protocol was previously reported [13]. However, in fixed protocols, the duration of progesterone exposure is prolonged, which has raised greater concerns regarding its potential impact. Nonetheless, the results of our study do not substantiate these concerns. In concordance with the literature [15–18], our results indicated that the number of oocytes retrieved, the maturation rate, and the number of blastocysts obtained were similar between the two protocols even GnRHa was used for trigger.

Although a recent study has demonstrated a more robust post-trigger LH response in flexible PPOS cycles compared to GnRH antagonist protocols—likely due to milder pituitary suppression by progestins such MPA—the evidence remains preliminary [13]. Most of these findings are based on limited patient cohorts, and thus the level of evidence is still low. Notably, while these studies focused on flexible PPOS protocols, our investigation was conducted using a fixed PPOS regimen, in which the duration of progestin exposure is longer. This difference in protocol design may potentially influence the hormonal environment during stimulation and the subsequent ovulatory response. However, despite theoretical concerns regarding extended progesterone exposure, our results do not support any adverse impact on oocyte yield, maturation, or embryo development. These findings underscore the importance of further large-scale, prospective studies comparing fixed and flexible PPOS protocols to clarify their differential effects and optimize clinical outcomes.

Another concern in PPOS cycles with GnRH agonist trigger is the potential delay in the ovulation process extending beyond 36 h. In the study by Xie et al. [19], it was demonstrated that exogenous progesterone administration could suppress endogenous LH levels and downregulate LHCGR expression in preovulatory follicles. Based on these findings, the authors speculated that ovulation might be postponed under such conditions. However, in our clinical practice, oocyte retrieval was consistently performed 36 h after the GnRH agonist trigger and we observed that the number of oocytes retrieved, oocyte maturation rate, and the number of blastocysts obtained were comparable between patients undergoing PPOS and those receiving a GnRH antagonist protocol. In addition, progesterone-mediated suppression is not limited to pituitary action, but also involves modulation of granulosa cell responsiveness to LH. While our study used a fixed-dose MPA protocol, future research should investigate whether flexible or lower-dose regimens lead to differential outcomes in LH responsiveness and ovulation timing.

This study has several notable strengths. First, it includes a large sample size, with 802 patients undergoing stimulation and 689 frozen embryo transfer (FET) cycles, which enhances the statistical power and generalizability of the findings. Second, strict inclusion criteria were applied—patients receiving a uniform starting gonadotropin dose of 300 IU, allowing for a more homogeneous population and reducing clinical variability. Third, this study compares two commonly used stimulation protocols under a uniform trigger approach using GnRH agonist, which is typically reserved for high responders due to its lower risk of OHSS. This ensures a relevant and clinically meaningful comparison in a population at risk for hyper-response. Fourth, multivariate regression analyses were used to control for confounding variables, lending robustness to the conclusions regarding embryo yield and live birth outcomes. Finally, the real-world nature of the data, drawn from routine clinical practice in a high-volume IVF center, enhances the external applicability of the findings.

Despite the valuable insights provided by our study, certain limitations should be carefully considered. First, the retrospective design precludes the establishment of definitive causal relationships and remains vulnerable to inherent selection bias, even though multivariate regression models were utilized to minimize confounding. Second, the single-center setting may limit the external validity of our results, and variations in clinical practice across centers may yield different outcomes. Third, while hormonal profiles on the trigger day were analyzed, the lack of serial LH and progesterone measurements during the stimulation phase limits our ability to fully elucidate the dynamic endocrine environment under PPOS protocols—especially the mechanisms by which progestins exert pituitary suppression. Moreover, although the GnRH agonist trigger is typically employed in high-responder populations due to its lower OHSS risk, our study did not include subgroup analyses based on ovarian reserve status. This prevents the identification of potential differences in protocol efficacy among normal and poor responders. Finally, the absence of cumulative live birth data and long-term reproductive outcomes restricts a more comprehensive evaluation of protocol efficiency over time. This design choice was intentional to reduce heterogeneity that may arise from differences in embryo quality, and patient attrition over time. While this approach allowed for a more controlled comparison between protocols, it limits the ability to assess the cumulative clinical success over multiple transfer attempts. Nevertheless, although cumulative live birth outcomes were not analyzed, our regression analysis of the number of usable frozen blastocysts showed no significant difference between the PPOS and GnRH antagonist groups, supporting the comparable embryologic efficiency of both protocols.

To address these gaps, future research should prioritize prospective, randomized controlled trials comparing fixed and flexible PPOS protocols in well-defined ovarian responder subgroups. Such studies should include detailed hormonal monitoring throughout the stimulation period, assess the timing and adequacy of the LH surge following GnRH agonist trigger, and evaluate whether different progestins or their dosing regimens differentially affect oocyte yield, quality, and subsequent embryo competence. Additionally, cumulative outcomes—such as total number of transferable embryos and live births across all FET cycles—should be reported to determine the true clinical utility of each stimulation strategy. These efforts will help refine clinical protocols and support a more individualized approach to ovarian stimulation in assisted reproduction.

In conclusion, despite the unclear mechanism by which progestins suppress the pituitary, our findings suggest that GnRH agonist triggering yields comparable oocyte numbers and pregnancy outcomes in both protocols. Nonetheless, further randomized trials are needed to confirm these results and assess cumulative outcomes.

## Data Availability

All data generated or analyzed during this study are available from the corresponding author upon reasonable request.
